# “You feel like you’re part of something bigger”: exploring motivations for community garden participation in Melbourne, Australia

**DOI:** 10.1186/s12889-019-7108-3

**Published:** 2019-06-13

**Authors:** Jonathan Kingsley, Emily Foenander, Aisling Bailey

**Affiliations:** 1 School of Health Sciences, Faculty of Health, Arts and Design, Swinburne University of Technology, 12 Wakefield Street (Swinburne Place West), Hawthorn, Victoria 3122 Australia; 2 School of Arts, Social Sciences and Humanities, Faculty of Health, Arts and Design, Swinburne University of Technology, 397 Burwood Road, Hawthorn, Victoria 3122 Australia

**Keywords:** Community garden, Motivations, Urban, Ecological model of health

## Abstract

**Background:**

Increased global urbanisation has led to public health challenges. Community gardens are identified as a mechanism for addressing socio-ecological determinants of health. This study aims to explore motives for joining community gardens, and the extent to which participation can be facilitated given barriers and enablers to community gardening. Such a study fills a gap in the public health literature, particularly in the Australian context.

**Methods:**

This paper presents findings from semi-structured interviews with 23 participants from 6 community gardens across Melbourne. Applying phenomenological, epistemological and reflexive methodologies and thematic analysis of the data, this study provides a snapshot of drivers of community garden participation.

**Results:**

Results were categorised into six enabling themes to participation. These themes revolved around (i) family history, childhood and passion for gardening; (ii) productive gardening, sustainability and growing fresh produce in nature; (iii) building social and community connections; (iv) community and civic action; (v) stress relief; and (vi) building identity, pride and purpose. Time costs incurred, garden governance and vandalism of garden spaces were among the barriers to community garden participation.

**Conclusion:**

Although an interest in the act of gardening itself may be universally present among community gardeners to varying degrees, the findings of this study suggest that motivations for participation are diverse and span a range of ancestral, social, environmental, and political domains. This study contributes exploratory insights on community garden motivations and sustained involvement across multiple urban sites in Melbourne (Australia). This study recommends extending this work by undertaking future quantitative research that can move from local case studies to a national guidelines on how to engage more people in urban agriculture activities like community gardening.

**Electronic supplementary material:**

The online version of this article (10.1186/s12889-019-7108-3) contains supplementary material, which is available to authorized users.

## Background

With the global urban population exceeding 54% [1] academic literature is increasingly considering the associations between urbanisation, socio-environmental changes (such as ecological destruction and disconnection from natural environments), and negative health consequences [2, 3]. Urbanised areas are “places where socio-environmental problems are experienced most acutely” [4]. Andersson and colleagues [5] associated urbanisation with living outside of “biophysical planetary boundaries” and suggested that engagement in green spaces/infrastructure can remedy environmental and health problems by reconnecting humans back to ecosystems.

A theoretical framework exploring the intersection between ecosystems, human and non-human health and wellbeing is the ecological model of health [6, 7]. There has been a proliferation of research fields that have evolved from this model, such as ecological public health and planetary health [8]. Community gardening has been identified as a way of improving health and wellbeing from an ecological public health perspective. When exploring this setting it is important to distinguish community gardening from other forms of gardening (e.g. school gardens) in that community gardening is communal and collective in nature and cuts across ages, genders and cultures. Participation in community gardening has been linked to economic and ecological benefits [9], and evidence indicates that such amenities improve health [10–12], wellbeing [13, 14], social [15, 16], and sustainability [3, 17–19] outcomes at an individual and planetary scale. This is because community gardens and other forms of gardening provide affordable and convenient fresh food, horticulture therapy and learning environments that improve academic performance, social interaction, and respite [13, 20–28]. Wells et al. [29] and Soga et al. [30] conclude that community gardens can address health inequalities. Specifically, community garden participation can have cognitive benefits for people living with dementia, providing opportunities “to live beyond the stigma and stereotypes associated with their conditions” [31]. Firth et al. [32] suggest that community gardening strengthens communities and mobilises people, but there is no universal consensus as to what extent this occurs.

Despite the lack of universal consensus on the potential for community gardening to promote community and collective action, it is widely recognised that they represent a cost-effective strategy for healthy public policy [14, 33, 34]. This is because community gardens reduce urban decay and food insecurities, address social and economic stressors, increase healthy food choices, promote regular exercise, social interaction and trust [18, 32, 34, 35]. As Shostak and Guscott [36] explain, community gardens “serve as a model for interventions that… “amplify” individual and community assets in support of public health”. The presence of community gardens in urban spaces can also present socio-environmental challenges, including tensions resulting from building/infrastructure development and urban community gardens raising property values [12]. As such, Barnidge and colleagues [37] and Turner [3] assert research is required to inform long-term and sustainable approaches to scaling up community garden initiatives.

A range of social, environmental, health and political motivations have been acknowledged in the literature around community garden participation. Social motivations revolve around strengthening community through enhancing social ties and civic engagement [14, 18, 38–40], with leading academics reporting positive associations between these motivations and increased social capital, resilience and cohesion [41–43]. Some commentators have suggested that these benefits have the scope to traverse cultural divides [43, 44]. Conversely, other studies have posited that community gardeners frequently align with others who share similar interests and social status, with social exchanges between gardeners rarely extending beyond garden settings [14, 15, 40]. Existing literature cites a range of environmental justice drivers for community garden participation such as: increasing environmental knowledge; improvement of food supply; neighbourhood pride; reconnection with nature; and reclaiming of neglected locations [4, 14, 18, 39]. Ghose and Pettygrove [45] define community gardens as sites for mobilising communities:

“enabling citizens to… participate in shaping their urban environments… spaces through which citizens can challenge dominant power relations and claim rights to the city… and resist local government policies”

Urban engagement in community gardens leads to economic gains due to positive impacts such as: sharing of resources; neighbourhood improvements; enhancing employability; and tackling food insecurity [39, 42, 43]. Such associations underpin the assertion that community gardens are beneficial for mental and physical health [14, 18]. These positive outcomes in turn lead to more inclusive socio-political environments, within which citizens can engage in urban citizenship to collectively reclaim land and enhance social equity [39, 41, 45]. In supporting community action, community gardens have been shown to increase leadership/decision-making skills and democratic values [39, 45]. Hence, as Crossan and colleagues [41] explain community gardens represent a form of Do-It-Yourself citizenship that encourage social relationships, human connectedness with nature and the constituents of effective political practices.

Political motivations for participating in community gardens vary. Some commentators cite aims to reclaim food systems, engage in ecological stewardship, and urban agriculture as motivations for participation [46]. Others frame community garden participation as a “resistance against poverty and hunger” and environmental degradation to address structural inequalities [47]. McClintock [46] observes that existing community gardens literature has increasingly claimed community garden models as a golden bullet to complex social, public health, ecological and community challenges. In response, McClintock [46] argues that: “[a] more critical camp of social scientists peels back this laudatory discourse… [demonstrating that] despite their progressive and radical intentions… [they] are neoliberal in their outcomes, or reformist at best, in that they continue to work within the capitalist logic of food systems”. Thornton [48] discusses this in the Australian context observing that community gardens struggle to co-exist in mainstream society with governments undervaluing their potential.

Drawing on phenomenological data, the present study was designed to understand the motives for joining and drivers for ongoing community garden participation. It builds on the author’s research [10, 15] which recommended scaling up qualitative studies of community gardens in Melbourne to compare and contrast perceptions of this activity across garden settings. As Turner [3] notes, “there is scant research looking at why people become, and stay, involved in community gardens and their relationship to broader environmental concerns”. The present study responds to the call by academics who assert that there is a need to expand the geographical scope of community gardens research, in order to understand “different social and political contexts” beyond the United States of America [USA] [2, 49].

Upon closer examination gardening can take many forms and is a popular leisure activity with therapeutic benefits [13, 28, 50, 51]. Research suggests the existence of gender differences (likely reflective of gender conditioning) in gardening behaviour. For example, both Bhatti and Church [52] and Scott et al. [53] noted that gardens were sites where power and gender dynamics could be observed, given that gardens have historically been considered “masculine” places used for productive purposes, with women’s relationships to gardens generally situated as more recreational than functional. Organic gardening is an exception to this rule [53]. Armstrong [54] noted that women of high socio-economic status tend to spend more time gardening compared with men, while in lower socio-economic households, the time spent by men and women was comparable. Research indicates women have stronger attitudes toward environmental activism and engagement with nature [55].

Community gardens originated in the 1890’s, and were fundamental during World Wars in Europe and the Civil Rights movement in America as a means to supplement food in times of crisis [38, 44, 56]. In Australia, the first community garden was established in 1977 [15] and there are presently almost 600 gardens nationwide [57]. As Draper and Freedman [38] note, “throughout history, community gardens have come and gone in conjunction with the socio-economic climate of the country.” Community gardens are hard to define as they can be single plots or collective gardens of various settings, size, geographical location, governance structure, and function [4, 9, 14, 32, 45, 49]. The present study applies the broad definition of community gardening offered by Kingsley et al. [10], who describes it as “plots of land allocated to individuals to create gardens of their choice in a communal environment”. The terms allotment garden and community garden are often used interchangeably in literature, despite fundamental differences [23, 43]. Allotment gardening involves a piece of land allocated for personal use on a lease or rent basis, whereas community gardening involves a more collective and communal process [12, 53]. Some academics have abandoned the term community garden in favour of alternative labels such as “organised garden projects” [40]. Irrespective of terminology:

“The rational for community gardens are unclear… we do not know… to what extent participants are driven by potential social effects. The literature is not conclusive” [49]

What is clear is that community gardens provide a sense of belonging, as well as a place and identity for citizens [2, 39, 53]. Turner [3] identifies community gardening as a place-making activity where participants can experience a deep attachment, including a sense of belonging or even violation (if vandalism to gardens occurs). Cumbers and colleagues [42] explain that community gardens offer a space for a more active sense of place where multiple co-existing benefits are available and diverse views can come together to create new social relationships. Some scholars believe community gardens act as “third place” settings beyond home and work which are non-commercial, community-building, aesthetically pleasing and enhance social life across genders, cultures and ages [4, 40, 49]. The present study aims to explore motivations across garden settings, which in the literature has been recommended as a critical next step [49, 58] in order to move beyond the limitations of “small scale qualitative studies” localised to single community gardens [53]. The following study is the first peer-reviewed article exploring various community garden sites (*n* = 6) in Melbourne, Australia. This article significantly contributes to the emerging area of community gardening research internationally, which builds on Nettle’s seminal works in Australia [59].

## Method

The present research applies a qualitative approach as it offers an in-depth understanding of how individuals perceive community gardening [60, 61]. The research team aimed ‘to select information rich cases’ [62] to gain a better understanding of this topic with such an approach providing scope to respond to unexpected findings [63]. Ethics was approved for this qualitative research project from Swinburne University of Technology [SHR Project 2017/135].

Phenomenology is the philosophical discourse associated with studying how people make sense of the world around them and how life experiences may affect these perceptions and values [63]. In research it is difficult to disconnect from an individual’s experience, therefore, this study acknowledges that this exploration is in part informed by the researchers’ subjective perspectives. In this case such perspectives refer to how various population groups in Melbourne (Australia) perceive and participate in community gardening. Therefore, a phenomenological methodology was chosen to gather the data and to facilitate a better understanding of the structures of human consciousness [64].

The researchers aimed to apply this methodology by involving themselves in community consultation with gardeners and engaging in regular contact with 3000Acres [65]. 3000Acres is an urban agriculture advocacy group (acting as a peak body for community gardens in Melbourne). Further, the application of reflexivity was included involving the lead researcher diarising his perspectives after meeting gardeners and after consultations. This process allowed the researcher to better understand and describe participant experiences, and consequently an interpretivist epistemological perspective was also applied [63, 66, 67].

This study used the qualitative method of semi–structured interviewing. A 22 question interview guide (see sample questions in Additional file 1) was developed covering participants’ background, motivations, limitations, benefits and outcomes of this activity. Recruitment began through 3000Acres sharing the researchers’ invitation to participate with organisers of community gardens associated with this network. Snowball sampling was then employed in order to identify key stakeholders [62, 68]. Subject to consent by participants, the interviews were conducted face-to-face, audio recorded and transcribed verbatim. Prior to the interviews taking place, participants reviewed a Consent and Information Statement and a Consent Form was signed. Participants were offered the opportunity to view a summary of findings and to review their interview transcripts. Interview durations ranged between 45 min and 2 h.

Participants were allocated a pseudonym to protect their identities and that of the community garden. There were 23 participants in this study, 22 of whom were community gardeners from six community gardens across Melbourne, and one a representative from 3000Acres. Of the 23 participants, 17 were female and six male and all were of English-speaking background. Participants’ and community garden information is provided in Table 1. It is important to highlight that in an Australian, North American and Canadian context the term ‘community garden’ often refers to both community gardens and allotment sites because they offer both communal and individualised benefits [23]. The garden type was defined by the research team through reviewing descriptions provided by participants. The start date section of Table 1 shows that most participants were founding members or established within the garden setting.

**Table 1:** Community garden and participants information

Name	Year founded	Supporting organisation	Member #	# of plots	Plot Size	Garden type	Participant in study	Start date
Knox	1984	Knox City Council	120–150	124	~ 3 m × 10 m	Mix of communal & allotment gardening	4	1999 2010 2013 2013
Fareshare Garden	2015	FareShare Food Charity	900 volunteers involved in kitchen & garden	Communal land for Farshare Kitchen	~ 800 square metre [m^2^] garden	Community garden & kitchen	4	2005 in kitchen 2015 2015 2016
Condell St	2013	3000Acres	30	36	Industrial-strength plastic container on 250 m^2^ land	Mix of communal & allotment gardening	7	2013 2014 2014 2015 2015 2015 2016
Kensington	2005 (re-develop in 2014 due to contaminated soil)	Melbourne City Council	~ 30	52	32 plots (11 m^2^) 20 plots (23m^2^)	Community garden	2	2005 2014
Gordon St	2007	Yarra City Council	Not available	Multiple wicking beds	Not available	Allotment garden	2	2007 2013
Happy River	2017	Footscray Community Arts Centre	30–40	8	Café garden	Community garden	3	2017 2017 2017

Data was analysed using thematic analysis [69]. Once the researchers completed the transcript, they read each transcript a number of times to immerse themselves in the data and draw out common themes. Open and axial coding was used in this investigation [64, 68]. Coding was double-checked by the research team throughout the coding process to draw out the richest data possible and reduce bias. Participants did not articulate health and wellbeing factors explicitly as a driver for initial engagement in community gardening, however these factors were influential determinants in continued engagement. Data revealed that the direct health implications of gardening were distinct; thus the authorship team decided to explore this in another paper as it went beyond the scope of the present research. Results from the present study focus on the social, political and environmental factors driving community gardening participation.

## Results

“the proof is that people turn up every week… you don’t do that if you don’t love it” (Angela)

The study drew out themes looking at the reasons participants initially joined and continued community gardening, as these both relate to motivation. However it did not differentiate these themes specifically to each garden setting because it goes beyond the scope of this paper. Themes generated by our analysis highlighted that passion for gardening and political motives informed decisions to join community gardens. Some participants cited social reasons for joining, however this was more a driver for sustaining involvement. There were seven key themes identified:

- *Family, childhood and history: *The gardening experience was part of participants family upbringing and childhood that led them to be passionate about this activity.
- *Productive gardening:* The enjoyment of growing fresh food, being sustainable and connecting back to nature in urban spaces.
- *Building social and community connection: *The building of local connection through educating others, sense of belonging and relationships with like-minded people.
- *Community and civic action:* The ability to create a better society through, for example, environmental justice in urban settings.
- *Building sense of identity and ownership:* The sense of place, pride and belonging associated with participation.
- *Stress relief:* The escapism from stress associated with urban settings.
- *Barriers:* factors such as the perceptions that others were not meeting expectations, vandalism and disrespect of the community garden.

Although the six community gardens yielded consistent findings around the six enabling themes, there were unique findings in reference to *barriers to engagement*. Specifically, participants tended to focus on either lack of trust of government institution, community respect, or inconsistent contributions by and conflict between members of the gardens.

### Family, childhood and history

Participants acknowledged their main reason for joining a community garden was the act of gardening in and of itself. For some, this interest was based on personal history, with references made to a “lifelong” interest in gardening facilitated through parents and grandparents. The need to garden was partly nostalgic for some (e.g. Abigail stated: “I grew up in… [suburb] when it was all orchids and my idea of wellbeing is probably a green landscape”). Skye recollected helping her mother in the garden as a child, and drew associations between these experiences and community garden engagement:

“I can remember picking beans… we always had vegies from the garden… I met my husband… gardening is a very important part of our life and family”

Participants frequently recounted childhood experiences in nature and connections with others. Participants who disclosed these early childhood experiences in the natural environment linked it to their pro-sustainability attitudes and connection with nature. Often participants mentioned that their parents coupled this love of gardening with civic engagement, which made community gardens a perfect fit. For example, Louise fondly remembered:

“I grew up in a small country town. My parents were very involved in the community, on every committee you can imagine”

### Productive gardening: fresh food, sustainability and connecting with nature

The importance of having a productive garden that grew fresh produce was frequently cited for community garden participation. The satisfaction of eating one’s own food was discussed, with Skye explaining it’s “knowing where your food comes from, no food miles… fresh fruit and vegetables, in season”. Bradley explained that “it makes your food taste a hell of a lot better… [when] you’ve grown it yourself”. Other gardeners stated that they grew vegies to save money, not shop at supermarkets or avoid plastic wrapped produce. For many, connection to nature was a fundamental driver for participation. For example, Hannah described a need to get her “hands into the dirt”. Abigail emphasised that this longing to connect with nature was “innate”. Many participants perceived that community gardens were an asset that nurtured their mind to make the “city more liveable”. Angela elaborated:

“with gardening, you put something in the ground, you nurture it, and you get a result… a sense that I was contributing, that I was reconnecting… with the land and people…there is no better way for me to regain a sense of self and calm”

It was frequently mentioned by participants that they had no space to garden at home living in apartments or rental properties. Participants described wanting their own patch since gardening was a huge part of their life and some just wanted the “communal aspect”. For others it was about learning new skills and sharing knowledge around activities like composting.

### Building social and community connections: learning and engagement

The social aspect of community gardening was a major reason for continuing participation. Shannon stated that it was important when trying to acquire this knowledge “to be around other gardeners. Because it’s so hard learning out of books”. Others wanted to preserve this education for future generations as they were concerned citizens. Often participants acknowledged that community and subsequent social support was more important than the garden itself, regenerating wasted cityscapes and engaging marginalised communities. Catalina highlighted:

“to me it’s much more about community… I worry… about the world that my grandchildren are going to inherit”

For many it created a sense of community where they “clicked” and found “a sense of belonging” in the city and a neighbourhood initiative with other locals you would not meet otherwise. This fostered a sense of sharing experiences and friendships as Catalina explained “there’s sort of two aspects to the garden and one is this is my plot, the other is… people come together… [it] becomes our garden”. Many participants recognised this was because the garden brought like-minded people together with similar values and a shared interest and passion for something bigger than themselves. Belonging to a like-minded “community network” helped individuals “intellectually” (Kate) and was likened to the experience of “belonging to a family” (Bree).

For participants it had to do with an attachment, residency or even locality to a garden. For some it was a duty to look after the local community with Catalina explaining, “I do feel… the quality of life of this neighbourhood is in part my responsibility”. For others it was about growing friendships and diverse social connections like a micro-community. For retirees or new residents of a locality this was seen as essential; as David highlighted:

“I’ve recently retired… If I didn’t have a plot here, I’d probably be… sitting at home… it really gives me a focus… an opportunity to find people that I can relate to”

Community gardens were perceived as promoting social connectedness, as “it’s a very close-knit community” (Louise). Sometimes these connections were confined to the garden through transactions associated with watering, gardening advice, and harvesting, but frequently social connections extended beyond the garden setting leading to long lasting friendships. For some “that sense of desire for social participation has been more than met” (Yvette). As Sarah explained: “if you’re here, working away – often, people come up and go”, “What are you doing?”

### Community and civic action

“gardens are… important, in terms of community development… being able to access fresh, healthy… produce, especially for groups that are facing disadvantage… the more we can grow, the more they can access” (Louise)

Participants felt like they were neighbourhood activists. Louise explained “it also gives people that confidence to… act, rather than be passive… this gives people power… [not] through the normal channels of government”. Participants felt like they were creating a better society saving people money, building a “small-scale” community and seeing others benefit “who are struggling to get their next meal” (Douglas). Participants perceived that they were helping others. For example, Tilly reflected:

“I’m quite good at creating a community around myself… I know other people struggle with it… the community garden is a way for me to create that community for other people”

It was widely agreed among participants that environmental consciousness was a driver for initial participation and sustained involvement. Gardeners felt like they were “contributing to the improvement of the environment and food security” (Yvette). Participants frequently spoke of making “sustainable choices” as “grass roots” members of a broader urban agriculture, horticulture or organic movement. For example, Abigail commented “it’s terribly important that we start using urban spaces more constructively… [to] reform urban spaces… gardening is just incredibly important to our survival”. Angela asserted “it’s about reclaiming the land”. Community gardeners also raised awareness around respecting the earth, bringing down temperatures in cities and tackling climate change. Pearl commented “I’m incredibly concerned about climate change… I’d like to see people living more sustainable lives… I really want to build capacity… so that the community can speak for themselves”. Some participants believed that by having productive gardens, it was possible to address sustainability issues. Others were more sceptical, stating:

“the amount of harvest we get from the garden… wouldn’t really feed a mouse… composting is important from an environmental point of view” (Catalina)

In fact, composting was seen to strengthen leadership and collaborations between local members and community gardeners. For Kate, this process was viewed as life changing: “I’ve been working with the council representatives… I’ve come up with a community composting plan… for other community gardens… as a result… I’ve just been appointed to… [council] urban agricultural consultative committee… out of my one metre little plot… I couldn’t have anticipated anything like that.”

Most participants acknowledged being heavily involved in the community and volunteer sector. Community gardens often offered a space for people to engage locally. Sylvia stated “I don’t think there’s many places in our communities that have that sort of openness; where it’s different cultural groups” and age groups coming together. Some gardeners had prior experience in setting up community gardens and wanted to engage in this activity locally.

### Building a sense of identity and ownership

Participants often mentioned building a sense of identity through community gardening because it brings a unique and innovative group together. Feedback on how gardening participation influenced identity was diverse. As Skye pointed out this depends on circumstances: “gardening here to a retiree, it’s about socialisation and friendships. When you talk about my generation… it’s about food security”. Sylvia reflected on the diversity of motivations for community garden involvement: “I see some people wanting to access a green space… some want to connect with other community members... Other people come from a gardening background”. Participants believed community gardens brought “people together from all different walks of life” (Douglas).

Community gardens were noted to strengthen pride, purpose and satisfaction. Phil described a “sense of history” and “ownership” of his locality associated with garden involvement. Pearl reflected on how her involvement in a garden facilitated “feeling at home within the people in your area… you feel… valued”. For some participants this helped provided “structure… and no one would judge you… it has really helped me… get back into work” (Angela). Responses by participants indicate that garden involvement provided a sense of joy. Angela went on to say “you feel like you’re a part of something bigger… it’s a real delight to walk in the gates… and knowing that you have had a hand in it”. Participants felt a sense of responsibility, being “ambassadors… we’re here because we’ve been entrusted… to keep this space” (Skye). This led participants reflecting a sentiment echoed by Bradley:

“If I’d never had the chance to do it, I don’t know whether I’d… miss it. But if someone said… “You can’t have a garden anymore,” that would be upsetting”

### Escapism from urban stress

Participants frequently expressed that they “needed” community gardens because they offered a de-stressing environment that was serene and quiet at times and socially stimulating at other times. Some people mentioned that it allowed them to “cope so much better with stresses” (Pearl). Most participants would frequently go to relax, for example, Bradley stating “I’ll knock off work, and... I’ll swing by... it’s quite exciting, going to see how everything is going… you get a bit of a sense of achievement”. Others felt it allowed them to grow, with Douglas mentioning:

“sometimes I feel like I need to get away from people, and one of the best avenues for that is actually gardening… it’s just good to be away from your mind and just doing… repetitive sort of work”

### Barriers to community garden participation

A range of barriers to community gardening participation were discussed. Participants frequently cited time as a barrier to increased garden involvement because everyone has pressures with daily life. Conversely, some participants described feeling overcommitted to the garden. This led to frustration in the lack of engagement and sustained momentum. Catalina highlighted, “I’ve got plenty of things to do, it’s not really as if I want to drag a whole community along”. Theft and vandalism were cited as barriers to involvement. Disappointment was expressed by participants who were doing “something really public and worthwhile”, yet experienced “lack of respect” from other community members. For some this was frustrating: “my husband just wouldn’t go near the place… [he] was furious… I felt a bit annoyed because we used to do it together” (Abigail). Some people were conflicted: “having a more secure garden would be nice… but then that’ll take away this funny interaction… with people” (Hannah). Participants reflected on a range of additional challenges, such as: community members lodging complaints to council; insufficient volunteers; excessively long waiting lists to join, and insufficient marketing of the garden within the broader community:

“securing a site for a garden is incredibly difficult… you need to be really flexible… usually if you do get some land... It might be… shady… bad soil or it might not be long term… it might be more expensive and… less appealing for people” (Pearl).

Clashes of opinion, competition and communication were sporadically mentioned, with some people not necessarily coming “with the best of intentions… if someone’s being unreasonable, you have to be really diplomatic... I wish the space was just more straightforward” (Sylvia).

## Discussion

Consistent with recent academic discourse [9, 43, 70] this study has shown that while some level of interest in gardening may be universal among community gardeners motivations for participation can be highly diverse. These motivations (grounded in political, social, economic, environmental and ancestral determinants) are the focus of the present study. In studying motivations for community garden participation, this paper aimed to explore catalysts, factors that sustain interest, and enablers/barriers to participation in Melbourne (Australia). Such an exploration across different garden settings was an identified gap in literature to move beyond predominant single community garden and USA based studies [49]. Guitart et al. [2], recommended such an extension acknowledging, “future research should therefore assess greater geographical spread of gardens… clearly researchers have only touched upon a small fraction of the potential scope of community garden research”. The study identified distinct reasons for joining and maintaining community gardening participation. For example people joined because of their love of gardening and historical connection with this activity whereas social and community connections was a drive for continuing participation compared with connection to nature which seemed to be a driver for both across garden settings. This illustrates the diversity in motivations, and how a gardeners’ motivation for initial involvement can change and evolve over time.

Familial and nostalgic motivations for community garden involvement were often expressed by participants, who shared memories of parents or grandparents gardening during their childhood. These childhood memories motivated participants to engage with gardening, green spaces and have pro-sustainability and civic engagement values. These values are evident in the community garden literature, which highlights that key motivations for participation revolve around reclaiming food systems, environmental stewardship, reconnecting with nature and environmental knowledge to address social inequalities, marginalisation and environmental degradation [39, 46, 47]. Community gardening was frequently embraced as a form of ethical food production, with indication that the process of locally growing food offered a cheap strategy to tackle concerns about food miles, wasteful packaging, and reducing food expenditure. This is supported by literature that recognises environmental justice as a major driver of community garden participation [4, 14, 18, 39].

Nature connectedness featured as a strong motivator for community garden interest. The nature connectedness aspect of community gardening was perceived by some to fulfil an innate need and simultaneously offer stress reduction. This is consistent with Kingsley et al. [10] who claim that this innate connection to nature associated with community gardening is based on E.O. Wilson’s Biophilia Hypothesis [71] which reflects humans innate affiliation and love of nature.

For gardeners who lived in apartments or otherwise congested inner-city dwellings, involvement in community gardening fulfilled a longing for nature connectedness. Community gardens were therefore a mechanism for dealing with the increase in urban development and this was seen as critical given the limitations of access to green spaces and disconnection from the natural environment evident in cityscapes. Gardeners saw community gardens as havens they could escape to.

Escapism was identified as a key contributor to participant wellbeing, and was explored in more depth in a separate paper by the authors as it went beyond the scope of this paper. Nonetheless, this escape from urban pressures provided by community gardens created a sense of place that has been well documented in literature [4, 9, 13, 15, 31, 35, 42, 49]. This research provided evidence to support community gardens as a platform for addressing issues around urbanisation, disconnection from nature and to effectively apply ecological public health approaches at a community level.

Social and community connectedness was a theme commonly cited as a driver for sustained community garden involvement. Garden(s) provided sites for community engagement, and participants reflected on the ways in which social connections and garden communities became forces of collective impact, to effect changes such as environmental regeneration, community inclusion and food sharing. This upholds Crossan and colleagues [41] assertion that community gardens created a Do-It-Yourself place for citizenship.

Community gardens were noted to be places where people of diverse backgrounds and interests came together. Specifically, retirees reported community inclusion benefits such as community garden participation mitigating loneliness. This supports the contention within existing literature that there is a perception that community gardening increases social capital, resilience and cohesion [41, 42]. The closeness of garden communities was discussed, however there were varied perspectives as to whether social connections extended beyond the garden context. This reflects the current debate in academic discourse, where some commentators claim community gardens traverse cultural boundaries [43, 58] and others claim it is confined to the garden setting [15, 40]. Further research is required to explore whether this matters to community mobilisation, with some scholars claiming it does not [39].

Community gardening as a form of community giving, was also identified as a motivator for involvement. This included the finding that most gardeners were already active in community/ volunteer sectors and saw participation as another form of giving. The nature of community gardening being based on collective activities was indicated as sustaining gardeners’ interest. Gardeners conceptualised community gardens as sites for neighbourhood activism, with the capacity for mobilising a microcosm of people in ways that imbued a sense of hope and self-efficacy among community members against government structures. Such mobilisation via community garden is supported in the literature because it increases social ties and networks [32].

Gardening activities such as communal composting were cited as having strengthening effects on community connections, collaboration and enhancing leadership. Many gardeners explained pro-environmental attitudes as motivators both for initial community garden interest, and sustained involvement. This supports McVey et al.’s [43] exploration of community gardens in Edinburgh, within which political and environmental drivers (for example land reclaiming) were noted as the main reasons for sustaining participation. These settings provided a “grassroots”, like-minded community who worked together to tackle issues like climate change, urban renewal and place attachment. These informal group connections enhanced participants’ identity, ownership and cultivated a sense of feeling valued in a non-judgemental environment.

However, participants noted that time costs associated with garden involvement posed a barrier to gardening. Some gardeners expressed frustrations at a lack of respect for garden spaces, such as vandalism, while other gardeners noted that low membership numbers and lack of government support were barriers to community garden participation. This supports claims by Thornton [48] and McClintock [46] who discuss the undervaluing of community gardens in capitalist societies, with governments failing to acknowledge the potential benefits of this amenity. Further, interpersonal differences and competitiveness, were also noted as challenges.

Previous literature has identified that community gardens had the potential to be both an effective healthy public policy [30, 35, 72] and “golden bullet” public health intervention, but it is evident from this research that motivations for participation are complex and therefore may not be a simple solution to improving population health. Gardening in and of itself was seen as the main driver for participation. Such a point is relevant in the same way as public health interventions revolved around activities like sports or arts may be tailored to a segment of the population. Therefore, gardening will only attract people who are interested in such an activity and may not be relevant to the wider population.

In identifying all these themes the study had a range of limitations which need to be acknowledged. As most participants were established or founding members of their community gardens it is possible that this biased the representativeness of the views expressed by the sample and thus is acknowledged as a possible limitation of this study. More detail in future research is required to compare and contrast the community gardens with each other, and although we identified some differences in the responses of community gardeners we cannot definitively make this comparison. Thus, further research around who benefits, and motivations for the development of community garden interventions is a logical next step so that this activity can have a wider reach at a population level. Specifically, further research to understand potential barriers and distinct differences between gardens is required through, for example, comparative case studies.

Although the depth and detail provided by qualitative community gardening studies remains invaluable, such qualitative insights are likely to be significantly strengthened and further contextualised by quantitative research, that enables a more systematic approach to the examination of community gardening participation. This suggestion is supported within existing community garden literature [3, 37, 46, 73]. Other scholars have reiterated the point that long term and larger scale study designs are required [50, 74]. Surls [75] mentioned this needs to involve action research with more consistent research protocols, data gathering and analysis processes so that community gardening research can be compared and contrasted. This study came some way to addressing this and the recommendation outlined by the lead author a decade earlier calling for a study that explored community garden settings across urban Melbourne (Australia). Either way, the assertions of Ohly et al. [76] are relevant to this study in that “more convincing quantitative evidence is needed to promote gardening programs as public health interventions”. As Ghose and Pettygrove [45] concluded, it will take considerable effort to scale up individual community garden projects, but it is critical to ensure their grassroots impacts are highlighted beyond the garden setting for their sustainability. The authors propose a multi-staged approach to future community garden research in Australia which would involve mapping of community gardens to develop a typology, a co-designed quantitative measurement tool and the development of national guidelines aligned with international best practice to effectively engage more Australians in these settings.

## Conclusion

Understanding the motivations for involvement in community gardening is of great significance, as various identified motivations of community garden participation are likely to be a fundamental component of enhancing ecological public health in the future. However, if this is to be considered seriously by policy makers and the wider community, there needs to be a clearer narrative of the motives and drivers for participation. This study provides a snapshot of the drivers, barriers and enablers for participation in Melbourne community gardening initiatives responding, to an extent, to understanding this topic better. Nonetheless, what this study suggests is that engagement in community gardens enhances social and natural connectedness in urban settings to improve health and wellbeing and address socio-ecological determinants of health. This study proposes that a more rigorous and consistent approach to research in this space is required not only by academics but also by the community, government and practitioners to ensure that this narrative can be strengthened and supported adequately both locally and globally. This may require quantitative measures, robust theoretical consistency, and more effective qualitative action-based research with rich data being collected across a range of cultural and geographical settings to ensure such programs can adequately address the consequences of growing urbanisation.

## Additional file

Additional file 1: Example of semi-structured questions. (DOCX 13 kb)

## Data Availability

The datasets generated and analysed during the current study are not publicly available due to ethical guidelines around confidentiality but can be obtained from the corresponding author on reasonable request.

## Abbreviations

m^2^: Square metre
USA: United States of America
