# Supplementary material for: “You feel like you’re part of something bigger”: exploring motivations for community garden participation in Melbourne, Australia
Source: BMC Public Health. 2019 Jun 13;19:745. doi: 10.1186/s12889-019-7108-3 (PMC6567388; doi:10.1186/s12889-019-7108-3)
Supplement: Supplementary file 1 — Example of semi-structured questions. (DOCX 13 kb) [file 12889_2019_7108_MOESM1_ESM.docx]

Additional file 1

Example of semi-structured questions

1. How did you first get involved in community gardening?
2. How would you describe your involvement in this community garden?
3. What attracted you to community gardening?
4. Did you hope to gain something in particular through being involved?
5. Have any of the things you hoped to gain through your involvement not come to be?
6. Has there been anything you have disliked about your involvement in the community garden?
7. Do you think there is anything that could be improved to enhance peoples’ experiences of the community garden?
8. Have any of the things you hoped to gain through your involvement come to be?
9. What do you feel as though you have gained through your involvement?
10. What do you feel is the most enjoyable part of your involvement?
